# The Promise and Limitations of Using Analogies to Improve Decision-Relevant Understanding of Climate Change

**DOI:** 10.1371/journal.pone.0171130

**Published:** 2017-01-30

**Authors:** Kaitlin T. Raimi, Paul C. Stern, Alexander Maki

**Affiliations:** 1 Gerald R. Ford School of Public Policy, University of Michigan, Ann Arbor, Michigan, United States of America; 2 Board on Environmental Change and Society, United States National Academies of Sciences, Engineering, and Medicine, Washington, District of Columbia, United States of America; 3 Norwegian University of Science and Technology, Trondheim, Norway; 4 Vanderbilt Institute of Energy and Environment and Vanderbilt Climate Change Research Network, Vanderbilt University, Nashville, Tennessee, United States of America; University of Washington, UNITED STATES

## Abstract

To make informed choices about how to address climate change, members of the public must develop ways to consider established facts of climate science and the uncertainties about its future trajectories, in addition to the risks attendant to various responses, including non-response, to climate change. One method suggested for educating the public about these issues is the use of simple mental models, or analogies comparing climate change to familiar domains such as medical decision making, disaster preparedness, or courtroom trials. Two studies were conducted using online participants in the U.S.A. to test the use of analogies to highlight seven key decision-relevant elements of climate change, including uncertainties about when and where serious damage may occur, its unprecedented and progressive nature, and tradeoffs in limiting climate change. An internal meta-analysis was then conducted to estimate overall effect sizes across the two studies. Analogies were not found to inform knowledge about climate literacy facts. However, results suggested that people found the medical analogy helpful and that it led people—especially political conservatives—to better recognize several decision-relevant attributes of climate change. These effects were weak, perhaps reflecting a well-documented and overwhelming effect of political ideology on climate change communication and education efforts in the U.S.A. The potential of analogies and similar education tools to improve understanding and communication in a polarized political environment are discussed.

## Introduction

Efforts to educate citizens about climate change have predominantly treated the topic as one of standard science education. The main goal has been to improve “climate literacy” by conveying established facts about the nature of climatic phenomena. Understanding of climate change is typically assessed by comparing people’s beliefs about climate change facts—including its human causes—to the scientific consensus [1–3]. Other measures assess respondents’ perceptions of the percentage of scientists who believe that anthropogenic climate change is underway, with answers in the high 90s considered accurate [4,5]. Conveying the latter percentage, which is actually information about scientists’ beliefs rather than about climate change itself, has been shown to be an effective method to increase belief in anthropogenic climate change [6,7].

We have argued elsewhere [8] that two other, less studied, objectives of climate change education are vital for citizens facing a future of climate change. One is to improve understanding that climate change presents various risks, many of which cannot be quantified with certainty but must still be considered. Education for this objective teaches that climate science has produced a mixture of solid knowledge and probabilistic knowledge, explains where uncertainty remains (including the trajectory of key risks), and clarifies processes by which scientists try to reduce uncertainties. The second educational objective is to inform people’s practical choices, including their policy preferences, in the face of climate change and its uncertainties. This is education for what is sometimes called decision support. Both objectives are important and currently lack adequate consideration in the literature.

### Educating the Public about the Unknowns of Climate Change

If the goal of climate change education is to create a public that is appropriately informed for decision making, then one key aspect that must be conveyed is that risk and uncertainty are both inherent in climate change. Treating the topic as one in which decisions can be based on established facts alone creates a mistaken impression of climate change risks. It also allows any attack on ideas portrayed as established fact to support the view that climate science is fundamentally in doubt, justifying inaction. People need to understand the progressive nature of climate change as well as the uncertainties to have informed debates about how to address the risks, costs, and benefits of possible actions and inaction.

The public is familiar with uncertain risks in many other domains, such as public health, terrorism, and earthquakes, which create possibility (but not the guarantee) that bad things may happen. People are also familiar with the idea that the exact timing and location of such events cannot be predicted with certainty. Experts in these arenas are often quite blunt with the public about these uncertainties [9–11].

### Informing Climate Decisions

Conveying uncertainty should be part of climate change education if its primary goal is to bring the public’s beliefs and understandings into line with scientific consensus. For informing climate-related decisions, education has the slightly different goal of helping citizens develop informed opinions about how to address climate risks. Informed choice requires understanding both of climate facts and of uncertainties: both what is known about climate change and what is potentially important, but uncertain. Many of the outcomes that motivate preemptive action on climate change most strongly (and are subject to the most intense debates) involve uncertain possibilities in the climate future.

Informing decisions does not imply bringing the beliefs of the public into line with any particular policy agenda. Differences of opinion are to be expected in dealing with any kind of risk. The goal is to support better-informed discussions and debates. This goal has been elusive in the U.S.A., where climate change is a highly polarized issue [3,12–15], and people tend to retreat to their own echo chambers when hearing and talking about it [16]. This polarization can undermine even the most promising methods of climate science communication [17]. It may prevent people from hearing about—and considering—the full range of proposed responses to climate change. Fully informed citizens, like climate policy experts, may well have differences of opinion and come to different conclusions about how best to proceed [18,19]. The goal of decision support is to help members of the public arrive at policy preferences and decisions that reflect the realities of the problem, even if their preferred solutions vary. Although evaluating success in this arena is trickier than assessing understanding of climate facts, people can report their understandings of key decision-relevant attributes of climate change.

### Important Decision-Relevant Attributes of Climate Change

We propose that to enable well-informed choices about responses to the risks of climate change, citizens should develop understanding not only of key climate facts, but also of key uncertainties and decision-relevant attributes of climate change. We recognize that some attributes will be more important for some people than others [20], but we propose the following elements of key understandings for the general citizenry. We believe that each of these elements reflects the scientific consensus on climate change. However, they do so at a more general level than the specifics of climate dynamics emphasized in typical efforts to promote climate literacy and are therefore more useful for informing people’s decisions and policy preferences.

1. Climate change is *anthropogenic* (human activity is responsible for a new kind and rate of climate change)
2. Climate change is *progressive* (it will have increasingly severe effects if unchecked)
3. Climate change is *hard to reverse*
4. Climate change can take the climate outside historical experience, such that *extreme events* of unprecedented severity may occur
5. There are *uncertainties* about how fast climate change will progress, where and when serious damage will occur first, in which environmental systems, and to what extent
6. The things people do that cause climate change also benefit people, so there can be *tradeoffs* in limiting climate change
7. The potential negative effects of climate change are linked, such that actions to slow or stop climate change *reduce the full set of risks*

Although acceptance of some of these elements of the climate science consensus may support policy preferences that favor actions to mitigate climate change (elements 2, 4, and 7), acceptance of others may support preferences against mitigation (elements 5 and 6).

### Simple Mental Models as Decision Guides

Simple mental models in the form of analogies may help convey these difficult ideas about climate change. Cognitive psychology research suggests that a good simple model should (1) be factual and not misleading, (2) use a familiar domain to explain the unfamiliar, (3) be novel enough to capture interest, and (4) allow for correct extrapolations based on understanding of the known domain [21,22]. Many such models have been used to help explain climate “facts.” For example, the analogy of Earth to a greenhouse makes it understandable why the planet’s surface temperature is so much higher than the temperature in outer space and identifies the role of atmospheric gases in retaining solar heat. Another simple model, comparing the atmosphere to a bathtub, helps explain the cumulative nature of carbon emissions [23]. These models may be useful for conveying certain aspects of climate dynamics, but not for the other pedagogical purposes we have identified.

This paper examines the potential of simple models for supporting not only climate literacy, but also the citizenship objectives of characterizing climate risks and uncertainties, and informing decisions given these uncertainties. For simple models to achieve all this, we have proposed that they should also (5) help users take into account uncertainty in climate projections, (6) recognize the need to consider options in the face of uncertainty, and (7) highlight unresolved issues in ways that provide space and conceptual guidance for public discourse among people who may initially disagree [8].

Although climate “facts” have been successfully conveyed through the use of simple mental models such as the bathtub and greenhouse analogies mentioned above, other research has been less promising. Likening climate change to a medical decision or to an engineering problem has not been more helpful than simple direct messages in increasing understanding of climate facts or the proportion of scientists who agree about anthropogenic climate change [5]. However, this work did not test whether analogies are helpful in other aspects of informing climate change decisions, such as the elements outlined above. Furthermore, the use of very short (one sentence) metaphors as was used in previous research [5] may not be sufficient to allow participants to fully grasp the ways in which a familiar domain (such as medical decisions) might act as a guide for other domains.

### Three Mental Models of Climate Change

A multitude of mental models could be used to explain climate change. We focus on three that capture some key attributes of the challenges of decision-making under uncertainty that climate change presents.

#### Medical disease

We expected a medical analogy to be most relevant to climate decision-making based on our own writing and the research of others [5,8]. Many medical diseases have decision-relevant attributes analogous to those of climate change: the risks are often caused or aggravated by human behavior (Element 1), the processes are often progressive (Element 2) and produce symptoms outside of the normal range of past experience (Element 4), and have uncertainties in prognosis of future events (Element 5). Also, the treatment of diseases often involves tradeoffs such as side-effects (Element 6) and the most effective approach is often to treat the underlying disease rather than alleviating symptoms (Element 7). In addition, some diseases, though not all, are hard to reverse (Element 3).

#### Disaster preparedness

Others have used analogies to disaster preparedness or insurance against disasters [24]. Although we do not believe that this analogy fits as well as the medical analogy, it has similarities. Among them are that disaster preparedness implies uncertainty about how and when disasters may occur (Element 5) and a need for some sacrifice involving effort or cost (Element 6). Disasters are inherently large and detrimental, therefore hard to reverse (Element 3). Some preparations—such as building with fire-resistant materials—aim to prevent the disaster in the first place rather than alleviating costs afterward (Element 7). However, preparing for a disaster does not necessarily entail that the disaster is human-caused (Element 1), or that the risks are progressive (Element 2) or unprecedented (Element 4).

#### Court trial

Finally, we have included the analogy to a courtroom trial in which lawyers on competing sides argue about facts to influence a decision. This analogy is invoked implicitly by some opponents of climate change mitigation, who justify their position on the ground that there remains some scientific doubt. We believe this analogy is less helpful than the others, as it puts people in the mindset of judging the existence of climate change rather than considering options for action. However, it does embody several decision elements. A courtroom trial implies a human cause to a problem (or crime) (Element 1) and an event that has already occurred and is thus difficult to reverse (Element 3). It may also imply some sense of progression, analogous to a defendant’s likelihood of recidivism (Element 2). But given that trials usually deal with past rather than future harm, the analogy does not evoke issues of unprecedented future harm (Element 4), uncertainties of future harm (Element 5), tradeoffs in preventing those harms (Element 6), or treating the underlying issues rather than deciding the fate of the current defendant (Element 7).

### Current Research

This research tests whether analogies can help educate people on uncertainty management, as well increase climate literacy as examined in previous research [5]. We tested the use of analogies against a standard climate-information message. In Study 1, we presented citizens with the three simple mental models above in the form of analogies, presented within longer messages that contain the seven key elements of climate understanding outlined above. We expected that analogies would help people understand the role of each of these attributes in climate change, and thus be better prepared to make and participate in decisions about how to address climate risks. Study 2 sought to replicate Study 1 and test whether changes to these decision-making elements result in changes to policy support. Finally, an internal meta-analysis was conducted to reconcile differences in results between the two studies and estimate the overall strength of effects.

Given the intense political polarization surrounding climate change in the U.S.A. [14], we also tested whether the effects of simple mental models varied by political ideology. Effective analogies might induce conservatives to attend to the need for decisions under uncertainty. Or, analogies might move liberals to move from general belief that a problem exists to considerations of what to do about it.

## Study 1 Methods

### Participants

Participants were 400 U.S. adults recruited via Amazon’s Mechanical Turk (MTurk) [25] in June of 2015, in exchange for $1.00. Participant demographics are shown in S1 Table.

### Procedure and Measures

All materials were administered using Qualtrics software. All study materials and procedures were approved by the Vanderbilt University Institutional Review Board.

#### Experimental manipulation

After providing written consent, participants read one of four passages describing climate change (see S1 Appendix). Three passages of approximately 350 words in length used analogies to explain climate change and decisions about potential climate action. The first condition, Medical Analogy (MA), described climate change as akin to a medical disease, with readers playing the role of the patient’s guardian. The second, Disaster Preparedness Analogy (DA), described preparing for climate change as akin to protecting a house against disasters like flood and fire. The third condition, Trial Analogy (TA), used the analogy of a courtroom trial for which readers imagined themselves as jury members. Participants in a fourth (control) condition read a passage about climate change in which no analogies were used.

#### Reading comprehension check

Reading comprehension was tested by asking, “In the passage you just read, was climate change described as being like any of the following?” with response options of (1 = *medical disease*; 2 = *courtroom trial*; 3 = *home protection*; 4 = *space flight*; 5 = *N/A none of the above*).

#### Self-reported helpfulness of passages

After reading a passage, participants were asked how much it helped them think about climate change (1 = *not at all* to 5 = *very*).

#### Climate science literacy

Climate science literacy was assessed by having participants indicate which of several causes of climate change are true and which are false, using the measure from Guy et al. [1]. Participants also estimated the percentage (0–100%) of climate scientists who believe that climate change is occurring and caused mostly by human activity.

#### Decision-relevant beliefs about climate change

Participants reported their level of agreement (1 = *strongly disagree* to 5 = *strongly agree*) with statements that mapped onto the seven decision-relevant attributes of climate change previously identified.

Two statements related to anthropogenic climate change formed a composite (Element 1): “The climate is changing” and “Climate change is caused in large part by human activities” (α = .77). The idea that climate change is progressive (Element 2) was assessed with the item “Climate change will get worse if we don’t do something about it.” Element 3 was measured with the statement, “Climate change is hard to reverse.” The statement, “Climate change may cause catastrophes, such as storms, floods, and wildfires greater than ever seen before” captured the idea that climate change will cause extreme unprecedented events (Element 4). The uncertainty of climate change’s exact effects (Element 5) was measured with the item, “It is difficult to predict when and where serious damage from climate change will occur.” Beliefs about tradeoffs (Element 6) were assessed with two items: “The things that people do that cause climate change also promote human comfort and convenience” and “Stopping climate change would be very disruptive to society.” A composite of these two items was considered, but they did not form a reliable scale (α = .26), and thus were analyzed separately. Finally, Element 7—the importance of mitigation (rather than just adaptation)—was assessed with the item “It is important to reduce the causes of climate change because that reduces all the negative effects—other actions only deal with one effect at a time.”

#### Demographics and political ideology

Participants reported demographic characteristics including age, gender, and race. They also indicated their political affiliation (1 = Republican, 2 = Republican-leaning independent, 3 = Independent, 4 = Democratic-leaning independent, 5 = Democrat) and overall political ideology ranging from 1 = *very conservative* to 5 = *very liberal*.

## Study 1 Results

### Reading Comprehension Check and Missing Data

Participants who failed the reading comprehension check were removed from the dataset (*n* = 47); only the remaining 353 participants are included in the following analyses. A disproportionate number of participants in the TA condition failed the reading comprehension, leaving fewer in that condition (*n* = 76) than in the other conditions (MA = 96; DA = 89; control = 92). All missing data for the remaining participants were deleted pairwise. As shown by the degrees of freedom for each regression in Tables 1 and 2, no more than three participants had missing data for any analysis.

**Table 1:** Climate literacy measures by political orientation, condition, and their interaction.

	Study 1					
	Climate literacy			Scientific consensus		
Predictor	*b*	*SE*	*sr*^2^	*b*	*SE*	*sr*^2^
MA	0.47^†^	0.27	.01	5.25	2.89	.01
DA	-0.20	0.28	.00	2.71	2.95	.00
TA	0.10	0.29	.00	6.29*	3.07	.01
*Step 1 F*	2.16^†^			1.75		
*Step 1 R*^*2*^	0.02			.02		
Ideology	0.59***	0.10		5.69***	1.02	.08
*Step 2 F Change*	38.56***			30.82***		
*Step 2 R*^*2*^ *Change*	.10			.08		
MA x Ideo.	0.05	0.27		-5.28^†^	2.87	.01
DA x Ideo.	0.33	0.27		-4.32	2.90	.01
TA x Ideo.	0.10	0.27		-2.19	2.93	.00
*Step 3 F Change*	0.61			1.33		
*Step 3 R*^*2*^ *Change*	.01			.01		
*Mean*	3.80			78.59		
*SD*	1.85			19.87		
	Study 2					
	Climate literacy			Scientific consensus		
Predictor	*b*	*SE*		*b*	*SE*	
MA	-0.12	0.30		-0.54	3.35	
*Step 1*	0.16			0.03		
*Step 1 R*^*2*^	.01			.00		
Ideology	0.67***	0.13		5.92***	1.46	
*Step 2 ΔF*	28.93***			16.57***		
*Step 2 ΔR*^*2*^	.13			.08		
MA x Ideo.	-0.07	0.25		-1.35	2.93	
*Step 3 ΔF*	0.08			0.21		
*Step 3 ΔR*^*2*^	.00			.00		
*Mean*	3.83			76.65		
*SD*	2.03			22.83		

^†^*p* < .10;

*p < .05;

***p < .001

df for Study 1: Step 1 (3,349), Step 2 (1, 348), Step 3 (3, 345)

df for Study 2: Step 1 (1,188), Step 2 (1, 187), Step 3 (1,186)

MA = Medical Analogy condition, DA = Disaster Preparedness Condition, TA = Trial Analogy

Condition, Ideo. = political ideology

Multicollineary tests (VIF and Tolerance) were with acceptable ranges (< 10 and > .10, respectively).

**Table 2:** Study 1: Decision-relevant elements of climate change by political orientation, condition, and their interaction.

Predictor	Element 1: ACC^1^			Element 2: Progressive^2^			Element 3: Hard to reverse^1^			Element 4: Unprecedented^1^		
	*B*	*SE*	*sr*^2^	*B*	*SE*	*sr*^2^	*b*	*SE*	*sr*^2^	*b*	*SE*	*sr*^2^
MA	0.21	0.12	.01	0.19	0.14	.00	0.23^†^	0.13	.01	0.29*	0.13	.01
DA	0.03	0.12	.00	-0.07	0.15	.00	0.06	0.13	.00	0.09	0.13	.00
TA	0.10	0.13	.00	0.05	0.15	.00	0.14	0.13	.00	0.29*	0.14	.01
*Step 1 F*	1.21			1.19			1.27			2.33^†^		
*Step 1 R*^*2*^	.01			.01			.01			.02		
Ideology	0.38***	0.04	0.21	0.42***	0.05	.18	0.12**	0.05	.02	0.34***	0.05	.14
*Step 2 ΔF*	96.28***			77.06***			7.31**			58.42***		
*Step 2 ΔR*^*2*^	.21			.18			.02			.14		
MA x Ideo.	-0.28**	0.11	.01	-0.31*	0.13	.01	-0.04	0.13	.00	-0.32**	0.13	.02
DA x Ideo.	-0.05	0.11	.00	0.05	0.14	.00	0.06	0.13	.00	-0.04	0.13	.00
TA x Ideo.	-0.10	0.11	.00	-0.15	0.14	.00	0.23	0.13	.01	-0.30*	0.13	.01
*Step 3 ΔF*	0.02^†^			2.92*			1.70			3.47*		
*Step 3 ΔR*^*2*^	.02			.02			.01			.03		
*Mean*	4.17			4.19			4.10			4.15		
*SD*	0.81			0.98			0.86			0.91		
Predictor	Element 5: Uncertainties^3^			Element 6a: Comfort^1^			Element 6b: Disruptive^3^			Element 7: Mitigation^1^		
	*B*	*SE*	*sr*^2^	*B*	*SE*	*sr*^2^	*b*	*SE*	*sr*^2^	*b*	*SE*	*sr*^2^
MA	0.00	0.13	.00	0.17	0.12	.01	-0.17	0.15	.00	0.31*	0.13	
DA	0.04	0.13	.00	0.22^†^	0.12	.01	-0.01	0.16	.00	0.31*	0.13	
TA	-0.33*	0.14	.02	0.24^†^	0.13	.01	-0.30^†^	0.16	.01	0.50***	0.14	
*Step 1 F*	3.12*			1.53			1.52			4.69**		
*Step 1 R*^*2*^	.03			.01			.01			.04		
Ideology	0.02	0.05	.00	0.13**	0.04	.02	-0.25***	0.06	.06	0.30***	0.05	
*Step 2 ΔF*	0.16			8.20**			20.43***			43.90***		
*Step 2 ΔR*^*2*^	.00			.02			.06			.11		
MA x Ideo.	0.09	0.13	.00	-0.35**	0.12	.02	-0.12	0.16	.00	-0.27*	0.12	.01
DA x Ideo.	0.10	0.13	.00	-0.16	0.12	.00	-0.13	0.16	.00	-0.05	0.13	.00
TA x Ideo.	0.32*	0.14	.02	-0.24*	0.12	.01	-0.01	0.16	.00	-0.33**	0.13	.02
*Step 3 ΔF*	2.05			2.98*			0.39			3.28*		
*Step 3 ΔR*^*2*^	.02			.02			.00			.02		
*Mean*	3.89			3.91			2.90			3.89		
*SD*	0.89			0.82			1.06			0.90		

^†^*p* < .10;

*p < .05;

**p < .01;

***p < .001

^1^df: Step 1 (1,349), Step 2 (3, 348), Step 3 (3, 345)

^2^df: Step 1 (1,346), Step 2 (3, 345), Step 3 (3, 342)

^3^df: Step 1 (1,348), Step 2 (3, 347), Step 3 (3, 444)

MA = Medical Analogy condition, DA = Disaster Preparedness Condition, TA = Trial Analogy Condition, Ideo. = political ideology

Multicollineary tests (VIF and Tolerance) were with acceptable ranges (< 10 and > .10, respectively).

**Fig 1:**
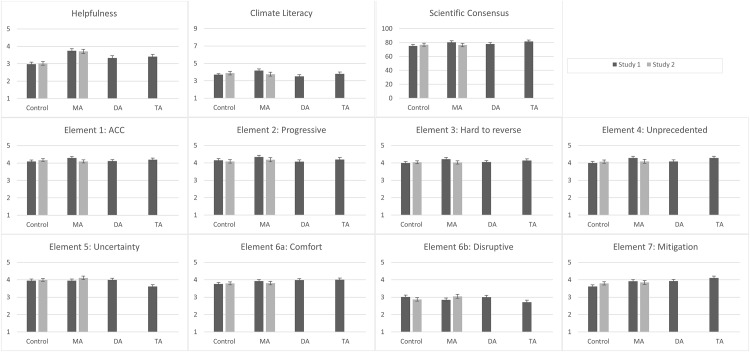
Means and standard errors of dependent measures by condition for Study 1 and Study 2.

The means for each dependent measure by condition are shown in Fig 1. Although our research questions focused on whether each analogy improved understanding above control levels, S2 Table also reports the results of analyses of variances with pairwise comparisons testing differences between the different analogies.

However, due to the importance of political ideology in climate change messaging, our focal analyses used hierarchical linear regressions (HLRs) to test the effects of condition, ideology, and their interactions on each of the dependent measures. Conditions were dummy-coded using the control as the referent. Political ideology was not mean-centered, but rather recoded so that the midpoint of the scale (moderate) was zero to create a meaningful intercept. All analyses included conditions in Step 1, political ideology in Step 2, and the interactions between ideology and each condition in Step 3.

### Self-Reported Helpfulness of the Passage

Participants in all three analogy conditions rated their passages as more helpful than control participants; this effect was particularly strong for the medical analogy (MA: *b* = 0.77, *t*(347) = 4.64, *p* < .001, *sr*^2^ = .06; DA: *b* = 0.35, *t*(347) = 2.09, *p* < .05, *sr*^2^ = .01; TA: *b* = 0.42, *t*(347) = 2.41, *p* < .05, *sr*^2^ = .02). Political ideology also affected reported helpfulness, with liberals rating the analogy passages as more helpful to their thinking about climate change than conservatives, *b* = 0.20, *t*(346) = 3.33, *p* < .001, *sr*^2^ = .03. Political ideology did not interact with any of the analogy conditions.

### Climate Science Literacy

Climate literacy descriptive statistics and regression estimates are shown in Table 1. Following the Guy et al. [1] method, the total number of incorrect causes of climate change that participants identified were subtracted from the total of correct causes (possible scores ranged from 0–9). The HLR found an effect only for ideology, with liberals demonstrating greater climate literacy than conservatives.

Participant estimates of the scientific consensus on climate change were also examined. Only 6.2% participants reported the exact 97% number used elsewhere [5], but 44.8% estimated a number between 90 and 100%. Both assignment to the trial condition and liberal ideology predicted higher levels of perceived scientific consensus.

### Decision-Relevant Beliefs About Climate Change

Condition, ideology, and their interaction were used to predict decision-relevant beliefs about climate change (Table 2).

#### Main effects

A number of condition effects emerged, but most were qualified by interactions. The exception was assignment to the DA condition, which was associated with greater belief in the importance of mitigation (Element 7), but not affected by ideology. Ideology was also a significant predictor of most decision-relevant outcomes. For example, liberal ideology positively predicted the belief that climate change is difficult to reverse (Element 3) and negatively predicted belief that mitigation would be disruptive (Element 6b). All other main effects were qualified by interactions, as outlined below.

#### Interaction of medical analogy and ideology

**Fig 2:**
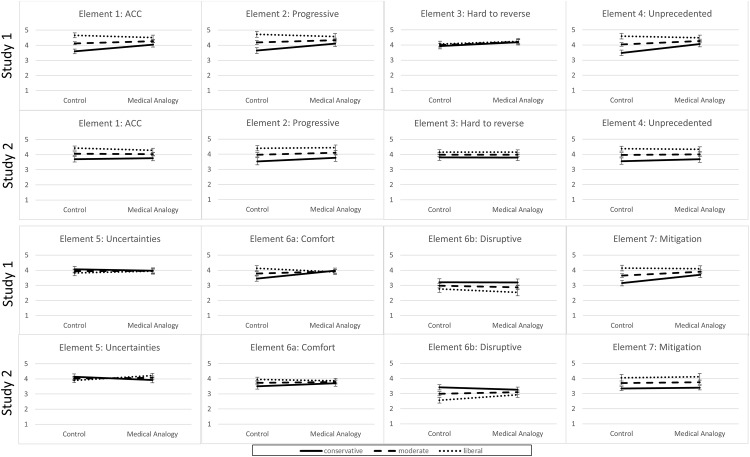
Decision-relevant outcomes by ideology and assignment to MA condition for Study 1 and Study 2.

A consistent pattern emerged in which ideology interacted with MA condition when predicting decision-relevant beliefs (see Fig 2). These interactions were generally driven by conservatives (and sometimes moderates) assigned to the MA condition endorsing decision-relevant elements more strongly than those in the control condition. Specifically, conservatives (and sometimes moderates) in the MA condition reported greater belief than controls that climate change is occurring and is anthropogenic (Element 1: *b*_Con._ = 0.45, *t*(346) = 2.87, *p* < .01), that it would progress if unchecked (Element 2: *b*_Con._ = 0.45, *t*(343) = 2.37, *p* < .05), that it is unprecedented (Element 4: *b*_Con._ = 0.58, *t*(346) = 3.21, *p* < .001; *b*_Mod_. = 0.24, *t*(346) = 1.95, *p* = .05), that emission-causing behaviors also provide comfort and convenience (Element 6a: *b*_Con_ = 0.53, *t*(346) = 2.99, *p* < .01), and that mitigation is needed to reduce the full set of risks (Element 7: *b*_Con_ = 0.55, *t*(346) = 3.04, *p* < .01; *b*_Mod_. = 0.26, *t*(346) = 2.13, *p* < .05). Liberals’ scores were not affected by the MA condition for any elements.

#### Other interactions

Despite the predominant findings that the MA condition x ideology interaction was most predictive of decision-relevant attributes, the interaction between ideology and assignment to the TA condition also predicted a few outcomes. Simple slope tests revealed that these interactions were also driven by conservatives and moderates. Conservatives in the TA condition reported greater belief in unprecedented consequences of climate change (Element 4: *b* = 0.54, *t*(346) = 2.87, *p* < .01) but also in the comfort and convenience of carbon-emitting behaviors (Element 6a: *b* = 0.47, *t*(346) = 2.54, *p* < .01). Being assigned to the TA condition also led conservatives and moderates to believe less strongly in the uncertainty of climate consequences (Element 5: *b*_Con_ = -0.74, *t*(345) = -3.67, *p* < .001; *b*_Mod_. = -0.37, *t*(345) = -2.73, *p* < .01), and more strongly that mitigation would be needed to reduce the full set of risks (Element 7: *b*_Con_ = 0.80, *t*(346) = 4.24, *p* < .001; *b*_Mod_. = 0.26, *t*(346) = 3.48, *p* < .001). No interactions emerged between ideology and assignment to the DA condition.

## Study 1 Discussion

Replicating past research, political ideology emerged as a strong predictor of reactions to climate messaging. Yet the current study extended beyond this finding to reveal the medical analogy as a promising way to achieve understanding of key decision-relevant elements of climate change processes, especially among non-liberals.

Our results were consistent with previous research on the use of analogies for climate change [5] in finding no effect of analogies on basic climate literacy or understanding of the scientific consensus. But participants reported that all three analogies were more helpful than the control description, with the medical analogy showing the most reliable effect. Also, analogies affected recognition of several decision-relevant attributes of climate change, and the type of analogy was an important factor. The disaster preparedness analogy only affected the results of one outcome (mitigation vs. adaptation). The results from the trial analogy were more mixed, with conservatives often becoming less accepting of some of the attributes of climate change (such as uncertainty) after reading that analogy.

The medical analogy had clear and consistent effects on understandings relevant to climate change decisions. For five out of the eight elements tested, conservatives (and to a lesser degree, moderates) who read the medical analogy reported greater agreement with these considerations than did those in the control group. Therefore, at least for the decision-relevant elements identified in previous research [8], the medical analogy was most consistently effective.

## Study 2 Methods

Study 1 demonstrated that using analogies for climate change, particularly a medical analogy, could encourage people to think about some of the decision-relevant attributes of climate change that are less commonly discussed in studies of climate literacy. Political liberals tended to recognize many of these elements irrespective of the framing of climate change, but using an analogy to a familiar context for decision-making under uncertainty increased recognition of these elements among conservatives. However, Study 1 did not test whether recognition of these decision-relevant elements translated into change in preferences about climate change action. For example, if conservatives who read about a medical analogy to climate change became more convinced of the need for mitigation (rather than simply adaptation), would this analogy also provoke support for mitigation policies? Study 2 set out to answer these questions.

### Participants

Two hundred American participants were recruited via MTurk in exchange for $1.00 in December of 2015. Sample demographics are shown in S1 Table. Written consent was obtained from all participants.

### Procedure and Measures

Given the lack of promising results for the disaster-preparedness and trial analogies in Study 1, only the medical analogy was tested in Study 2. Participants were randomly assigned to either the medical analogy (MA) or the control condition. A set of questions was added to assess participants’ policy preferences. Otherwise, all procedures and measures were identical to Study 1. This study was approved by the Institutional Review Board of the University of Michigan.

#### Policy preferences

Participants reported their preferences for various policy approaches to climate change. Participants first read a statement that “People have suggested different approaches to deal with the threats that we hear come from climate change.” They then used a 7-point bipolar scale to indicate which of two approaches was closest to their own view: “Wait to take action to reduce climate risks until we know that there are real dangers” or “Take action quickly to reduce the use of fossil fuels and other activities that contribute to climate change.”

Participants were also asked how much they supported each of nine policies that have been proposed to mitigate climate change (1 = *strongly oppose* to 5 = *strongly support*). The policies listed were: “Signing of an international treaty to reduce emissions from the U.S. and other countries”; “Requiring car manufacturers to increase the fuel efficiency of their vehicles”; “Increasing subsidies for renewable energy such as wind and solar power”; “A tax on fuel, based on the amount of greenhouse gases emitted by that fuel”; “Requiring appliance manufacturers to increase the efficiency of energy-using appliances”; “Building more nuclear power plants”; “Encouraging individuals to use less energy in their home and vehicles”; “Reducing emissions of large electric generating facilities and factories”; and “Setting a limit to greenhouse gas emissions with a system such as "cap and trade”.”

## Study 2 Results

### Reading Comprehension Check and Missing Data

Ten participants were removed from the dataset for failing in reading comprehension, leaving a total *N* of 190. None of the remaining participants had missing data.

Dependent measures were assessed with the same analytic strategy as in Study 1. Means are shown in Fig 1.

### Self-Reported Helpfulness of the Passage

As in Study 1, both assignment to the medical analogy condition and liberal ideology were associated with perceived helpfulness of the passage in thinking about climate change (MA: *b* = 0.69, *t*(188) = 4.21, *p* < .001, *sr*^2^ = .09; Ideology: *b* = 0.19, *t*(187) = 2.59, *p* < .01, *sr*^2^ = .03). Also replicating Study 1, the interaction was not significant.

### Climate Science Literacy

All climate literacy results are shown in Table 1. As in Study 1, liberals scored higher in climate literacy than conservatives and the medical analogy had no effect on literacy. Reported perceptions of the scientific consensus on anthropogenic climate change were similar to Study 1 results: 4.7% of participants estimated the precise 97% number, and 44.2% guessed a number between 90 and 100%. As in Study 1, liberals estimated greater consensus than conservatives, and neither condition nor the interaction of condition and ideology affected this belief.

### Decision-Relevant Beliefs About Climate Change

Responses regarding the seven decision-relevant attributes of climate change are summarized in Table 3 and interactions between ideology and medical analogy condition are shown in Fig 2.

**Table 3:** Study 2: Decision-relevant elements of climate change by political orientation, condition, and their interaction.

Predictor	Element 1: ACC		Element 2: Progressive		Element 3: Hard to reverse		Element 4: Unprecedented	
	*b*	*SE*	*b*	*SE*	*b*	*SE*	*b*	*SE*
MA	-0.08	0.12	0.10	0.15	-0.01	0.13	0.01	0.15
*Step 1 F*	0.43		0.41		0.01		0.00	
*Step 1 R*^*2*^	.00		.00		.00		.00	
Ideology	0.28***	0.05	0.34	0.06	0.15**	0.06	0.33	0.06
*Step 2 ΔF*	32.89***		28.06***		7.62**		28.33***	
*Step 2 ΔR*^*2*^	.15		.13		.04		.13	
MA x Ideo.	-0.09	0.10	-0.09	0.13	0.01	0.11	-0.07	0.12
*Step 3 ΔF*	0.90		0.48		0.00		0.36	
*Step 3 ΔR*^*2*^	.00		.00		.00		.00	
*Mean*	4.14		4.13		4.03		4.08	
*SD*	0.80		1.04		0.85		0.99	
Predictor	Element 5: Uncertainties		Element 6a: Comfort		Element 6b: Disruptive		Element 7: Mitigation	
	*b*	*SE*	*b*	*SE*	*b*	*SE*	*b*	*SE*
MA	0.12	0.13	0.01	0.13	0.18	0.15	0.05	0.14
*Step 1 F*	0.84		0.01		1.45		0.12	
*Step 1 R*^*2*^	.00		.00		.01		.00	
Ideology	0.00	0.06	0.14	0.06	-0.27***	0.06	0.32***	0.06
*Step 2 ΔF*	.00		5.84*		17.49***		28.27***	
*Step 2 ΔR*^*2*^	.00		.03		.08		.13	
MA x Ideo.	0.23*	0.12	-0.12	0.12	0.23^†^	0.13	0.01	0.12
*Step 3 ΔF*	3.94*		1.14		3.25^†^		.01	
*Step 3 ΔR*^*2*^	.02		.01		.02		.00	
*Mean*	4.04		3.80		2.95		3.82	
*SD*	0.88		0.88		1.02		0.96	

^†^*p* < .10;

*p < .05;

**p < .01;

***p < .001

df for all regressions: Step 1 (1, 188), Step 2 (1, 187), Step 3 (1, 186)

MA = Medical Analogy condition, Ideo. = political ideology

Multicollineary tests (VIF and Tolerance) were with acceptable ranges (< 10 and > .10, respectively).

Only liberal ideology predicted the beliefs in anthropogenic climate change (composite α = .75: Element 1), that climate change is progressive (Element 2), that it is difficult to reverse (Element 3) that climate change events are unprecedented (Element 4), that the causes of climate change also have benefits (Element 6a), and that mitigation (rather than adaptation) is important for reducing the full set of climate change risks (Element 7). Furthermore, the more liberal a participant’s ideology, the less they thought climate change action would be disruptive (Element 6b).

#### Element 5

The HLR on uncertainties about the precise effects of climate change revealed no main effects for ideology or MA condition, in keeping with Study 1. However, there was a significant interaction between ideology and assignment to the MA condition. Specifically, liberals were more likely to agree that there are uncertainties about the precise effects of climate change if they had read the medical analogy, *b* = 0.31, *t*(187) = 1.93, *p* < .05. Moderates and conservatives were unaffected by the MA condition.

### Policy Preferences

Only ideology predicted participant preference for taking a wait-and-see approach to climate change (vs. immediate action), with liberals preferring more immediate action, *b* = 0.64, *t*(188) = 5.99, *p* < .001, *R*^*2*^change = .16. A composite was created of the nine mitigation policy support questions (*α* = .87). A regression showed that only ideology predicted this measure, with liberals more supportive of mitigation policies than conservatives, *b* = 0.27, *t*(186) = 5.83, *p* <.001, *R*^*2*^change = .15. Regressions testing each individual policy preference separately showed the same pattern. Neither condition nor the interaction predicted any of the policy preference outcomes.

## Study 2 Discussion

Contrary to Study 1, Study 2 found almost no effect of a medical analogy on recognition of the decision-relevant elements of climate change. It also found no effect of the analogy on policy preferences. The only major replications in Study 2 were the findings that political ideology strongly predicted all climate change beliefs and that the medical analogy was considered helpful to understanding, regardless of political ideology. Study 2 also replicated Study 1 in showing that the medical analogy did not affect understanding of climate literacy facts.

Only one decision-relevant aspect of climate change was affected by the medical analogy in Study 2: uncertainty about climate change’s precise effects. Liberals (but not moderates or conservatives) were more convinced of these uncertainties after learning about climate change via a medical analogy. This effect did not appear in Study 1, suggesting that this finding is also less than robust.

The failure to replicate the effects of the medical analogy on beliefs was unexpected, given that the materials and procedure in Study 2 were identical to Study 1 except for additional measures added to the end of the questionnaire. Although we did not have a priori hypotheses for these disparate findings, one possible explanation is the months in which these data were collected (June, 2015 for Study 1 and December, 2015 for Study 2). Ambient temperature and weather have been shown to affect climate change beliefs [26,27], and thus the warmth during June may have made conservative participants more open to climate change information than were the participants in the cold of December. Another possible factor was the 2016 U.S. presidential campaign, which had picked up intensity by December of 2015 and may have increased the salience of political ideology.

Whatever the reason for the differences in statistical significance, the results for the two studies were often trending in the same direction. Therefore, we tested the overall effect of the medical analogy by combining the results of the two studies in an internal meta-analysis.

## Internal Meta-Analysis Methods

Meta-analysis is useful for synthesizing results from multiple studies, particularly when focusing less on statistical significance and more on the overall strength of effects [28]. This is particularly important as *p-*values can be unreliable across studies [28]. We therefore conducted a meta-analysis to determine overall effect sizes across the two studies. Specifically, we computed effect sizes of assignment to the medical analogy condition, and the interaction between medical analogy (vs control) condition and political ideology, on each of the decision-relevant elements of climate change. A separate series of meta-analyses computed the effect sizes for the simple slopes for conservatives and liberals separately.

### Participants

To facilitate comparisons between studies, only participants from Study 1 assigned to the medical analogy or control conditions were included in these analyses.

### Meta-Analytic Approach

Meta-analysis was conducted with the STATA 14 [29] metan command. All effects were translated into Cohen’s *d*s, either through relevant *M*s and *SD*s (comparisons of the main effect of the medical analogy vs control condition) or the *t*-values associated with regression predictors (either the relevant interactions or simple slopes). Standard errors were then computed for these effect sizes, and weighted, pooled effect sizes were generated across the two studies for each relevant set of analyses. We used random-effects models given the desire to generalize beyond the current samples [30]. We used Cohen’s [31] guidelines for the interpretation of effect sizes; *d* = .20 is considered a small effect size, *d* = .50 is a medium effect size, and *d* = .80 is a large effect size.

## Meta-Analysis Results and Discussion

### Main Effect of Medical Analogy Condition

Main effects of reading about climate change in terms of a medical analogy on decision-relevant beliefs were small (all *d*_+_s < .2; see Table 4). These effects were all positive, indicating that to the extent that a medical analogy alters people’s thinking about climate change, it does so in ways consistent with these elements.

**Table 4:** Internal meta-analysis of Studies 1 and 2. Main effects of medical analogy (vs control) predicting decision-relevant beliefs, interactions of medical analogy and political ideology, and simple slopes for conservatives and liberals of assignment to the medical analogy condition.

Element	Main effects of MA condition			Interactions of MA and ideology		
	Study 1 *d*	Study 2 *d*	Combined *d*_+_ (*CI*)	Study 1 *d*	Study 2 *d*	Combined *d*_+_ (*CI*)
Element 1	.25	-.10	.08 (-.26, .42)	-.37	-.14	-.25 (-.48, -.03)
Element 2	.20	.09	.15 (-.06, .35)	-.34	-.10	-.22 (-.46, .02)
Element 3	.27	-.02	.13 (-.15, .40)	-.29	.01	-.14 (-.43, .15)
Element 4	.34	.01	.17 (-.15, .50)	-.39	-.09	-.24 (-.54, .06)
Element 5	.00	.13	.07 (-.13, .27)	.11	.29	.20 (-.01, .40)
Element 6a	.20	.01	.11 (-.09, .31)	-.43	-.16	-.29 (-.57, -.02)
Element 6b	-.16	.18	.01 (-.32, .34)	-.11	.26	.07 (-.30, .44)
Element 7	.33	.05	.19 (-.08, .47)	-.30	.02	-.14 (-.45, .17)
Element	Simple slopes for conservatives			Simple slopes for liberals		
	Study 1 *d*	Study 2 *d*	Combined *d*_+_ (*CI*)	Study 1 *d*	Study 2 *d*	Combined *d*_+_ (*CI*)
Element 1	.42	.06	.24 (-.12, .59)	-0.03	-.15	-.09 (-.29, .11)
Element 2	.36	.15	.25 (.05, .46)	-.01	.03	.01 (-.20, .21)
Element 3	.18	-.01	.08 (-.12, .29)	.19	.00	.10 (-.11, .30)
Element 4	.50	.08	.29 (-.11, .69)	.04	-.03	.00 (-.20, .21)
Element 5	-.08	-.15	-.12 (-.32, .09)	.07	.28	.18 (-.03, .38)
Element 6a	.46	.14	.30 (-.02, .62)	-.08	-.08	-.08 (-.28, .12)
Element 6b	.02	-.10	-.04 (-.24, .16)	-.14	.30	.08 (-.35, .51)
Element 7	.41	.03	.22 (-.16, .60)	.09	.06	.08 (-.13, .28)

### Interaction of Condition and Political Ideology

The interaction of MA condition with political ideology mainly produced small effects. The largest effect sizes were observed for Elements 1, 2, 4, 5, and 6a. Fig 2 suggests that for each of these elements, less political polarization was found for people in the MA condition than in the control condition. For four of these elements, this decreased polarization was due to conservatives’ stronger endorsement of the attributes following the medical analogy. The exception was Element 5, in which liberals who read the medical analogy were more aware of the uncertainties in climate science.

### Simple Slopes for Conservatives and Liberals

Because the interaction effects in Study 1 were driven by changes among conservatives, the effect sizes for these simple slopes were computed again using meta-analysis. Small but positive effects emerged for Elements 1, 2, 4, 6a, and 7: Conservatives in the MA condition (as compared to the control) indicated stronger belief in anthropogenic climate change, stronger beliefs that climate change is progressive and unprecedented, greater belief that the causes of climate change also have benefits, and stronger belief that mitigation reduces the full set of risks. Thus, as suggested by Fig 2, the lessened polarization found among those in the MA condition was largely due to conservatives endorsing beliefs more in line with liberals.

Although fewer significant effects emerged for liberals, we used a meta-analysis to test the overall effect sizes for these simple slopes as well. The only effect that approached significance was Element 5, suggesting that liberals became more aware of uncertainties in climate science after reading the medical analogy.

## General Discussion

We identified seven decision-relevant attributes of climate change on the basis of a priori analysis [8]. We examined perceived helpfulness of the analogies, and their effects on climate “literacy” measures, beliefs about the extent of scientific consensus, and policy preferences related to climate change. Our main interest was in the effects of characterizing climate change via simple mental models using analogies from familiar contexts involving decision making under uncertainty that might make several key decision-relevant attributes of climate change more accessible. Our hope was that analogies that embodied these key attributes would better enable people to recognize these elements of climate change, and thus better comprehend the phenomenon, think through the risks it might present, and develop a more solid basis for making personal and political choices. Across the two studies, we found some evidence that the medical analogy was judged as helpful by respondents across ideological divides and increased individuals’ (particularly conservatives’) recognition of these decision-relevant aspects of climate change, though these effects were weak.

### Not All Analogies Are Created Equal

The medical analogy resulted in the majority of significant effects and changed the responses of conservatives consistently with respect to most of the key decision-relevant elements of climate change. The other analogies had weaker and less consistent effects. Respondents who read the trial analogy, particularly conservatives and moderates, were more likely to believe climate change will lead to unprecedented consequences and requires mitigation, but also that carbon-emitting behaviors provide comfort and convenience and that the timing and location of future damage from climate change are difficult to predict. The disaster-preparedness analogy had no effect on beliefs, except to produce an increase in the belief that mitigation is a vital form of response—a finding also observed with the trial and medical analogies. Unlike the other two analogies, the disaster preparedness analogy did not lessen the differences between conservatives and liberals in support for mitigation.

### The Usefulness of a Medical Analogy for Climate Change

We found that although the mental models we used had no effect on climate literacy or beliefs about the degree of scientific consensus, the medical analogy stood out as being seen as helpful and as affecting multiple climate-related beliefs. The most typical pattern of these effects was to shift the beliefs of conservatives (and to a lesser degree, moderates) toward the scientific consensus regarding several key decision-relevant characteristics of the climate change phenomenon, while leaving the beliefs of liberals largely unchanged. This pattern was observed with most of the beliefs examined, but not all. Effect sizes across the two studies suggested that non-liberal respondents became more convinced of the human causes of climate change, the progressive and unprecedented nature of climate change, and the need for mitigation to reduce the full set of risks. The medical analogy also led liberals to be slightly more aware of the uncertainties of climate projections. However, there were no effects on beliefs about the reversibility of climate change or the disruptive effects of mitigation.

Although promising, the effects of the medical analogy were generally weak. With issues as complicated and politically polarized as climate change, it may be too much to expect that any way of framing the issue in 350 words will lead to major shifts in understanding. Our studies replicated the well-established finding that political conservatives in the U.S.A. are more skeptical than liberals regarding many attributes of climate change that are consensually recognized in the scientific community [14]. Others have shown that many popular ways of framing climate change are ineffective in changing understanding among U.S. conservatives [17]. Thus, the finding that a medical analogy may lessen polarization in how conservatives and liberals view climate change is promising, even though the effects can best be interpreted as only suggestive.

We do not read our findings as suggesting that the medical analogy is a useful way to persuade conservatives to think more like climate activists. In line with our objective of improving nuanced thinking about climate decisions, we found changes that reduced polarization in other ways. For example, the medical analogy affected liberals by increasing their belief that many uncertainties still exist in climate change predictions. Furthermore, although the medical analogy helped respondents, especially conservatives, appreciate aspects of climate change that they would not otherwise endorse, it did not affect knowledge of climate “facts” or beliefs about the degree of scientific consensus. We also found in Study 2 that it did not change certain key policy preferences, particularly willingness to take a wait-and-see approach to policy in response to climate change or support for specific policies. We hesitate to draw conclusions from this non-finding because in Study 2 the medical analogy had such miniscule effects on the understandings that might be required to change policy preferences.

We conclude that the use of a medical analogy has promise for increasing people’s sense of ease in thinking about climate change as a phenomenon calling for decision making under uncertainty, for calling the attention of citizens to important decision-relevant aspects of the climate change phenomenon, and perhaps also for reducing the polarization of views in the U.S.A. along lines of political ideology. As already noted, this polarization has been highly resistant to past efforts to reduce it by educational means [17]. In that context, even weak effects such as those reported here are noteworthy. We suggest that the weak results may be due in part to the medical analogy being presented only very briefly. Further research should test the effects of more extensive exposure to the medical analogy. For example, it could be used in small-group or community discussion formats, or paired with other forms of decision support, such as computer programs that allow people to model consequences of climate decisions [32]. Further research could examine whether more extensive exposure to this analogy changes understanding more reliably or leads to changes in policy preferences.

### Limitations and Future Directions

As noted earlier, the overall effect sizes we observed were fairly small according to traditional guidelines [31], and most did not reach statistical significance in Study 2 or the internal meta-analysis. Power analysis using an expected *d* of .20 and with the desire for 80% power to detect a significant effect requires approximately 250 participants in each condition. Given these small effect sizes, future research in the area may require larger samples, at least if using null-hypothesis significance testing and not considering just effect sizes. Furthermore, if the differences in statistical significance between Studies 1 and 2 were, as we conjecture, due to the times of year and political environment in which they were conducted, future attempts to replicate climate change messaging should consider controlling for, or even more closely studying, these factors.

Another limitation of the current study was our use of an online convenience sample. Some of our findings were in line with those of nationally representative samples [5], and recent research suggests that participants recruited via MTurk respond similarly to nationally representative samples in regard to political issues [33]. Yet future studies should test whether the effects of analogies used in these studies replicate when used in more representative samples. The use of passages as long as the one in the current sample is often quite costly in survey research, especially in nationally representative samples, which presents challenges for using sample surveys with even more extensive elaborations of the medical analogy. Alternatively, the medical analogy could be useful in real-world settings where it could be presented in even more detail, interrogated, and deliberated. For example, this analogy could be used in the type of face-to-face conversations that have been sometimes shown to be remarkably effective at political persuasion [34].

Although our findings suggest that a brief encounter with the medical analogy is unlikely to change fundamentally the public’s basic understanding of climate mechanisms, they offer some cause for hope for this educational approach. By introducing novel ways of educating the public about climate change, such as decision-relevant analogies, climate communicators may be able to edge the public a bit closer to a deeper understanding of how to address such a wicked problem and an increased ability to engage in productive discussions despite ideological differences.

## Supporting Information

S1 Table Demographic breakdown of samples. (DOCX)

S2 Table Analyses of variance (ANOVAs) testing main effects of condition on dependent measures (Study 1). (DOCX)

S1 Appendix Experimental conditions. (DOCX)
