# Supplementary material for: The Promise and Limitations of Using Analogies to Improve Decision-Relevant Understanding of Climate Change
Source: PLoS One. 2017 Jan 30;12(1):e0171130. doi: 10.1371/journal.pone.0171130 (PMC5279784; doi:10.1371/journal.pone.0171130)
Supplement: S1 Table — (DOCX) [file pone.0171130.s001.docx]

**S1 Table. Demographic breakdown of samples.**

|  | Study 1 | Study 2 |
| --- | --- | --- |
| *N* | 353 | 190 |
| Age | 18-76; *M* = 34.41; *SD* = 11.80 | 18-69; *M* = 36.68; *SD* = 12.26 |
| Gender | 55% men | 48% men |
| Political affiliation |  |  |
| Democrat | 53.3% | 49.5% |
| Independent | 23.2% | 21.6% |
| Republican | 23.5% | 28.9% |
| Race |  |  |
| American Indian/Alaska Native | 0.3% | 0.5% |
| Asian/Pacific Islander | 6.5% | 8.4% |
| Black/African-American | 6.5% | 6.8% |
| Caucasian | 84.4% | 82.1% |
| Hispanic | 6.8% | 8.4% |

Note: Only includes participants who passed reading comprehension check.

Percentages do not always add to 100% due to rounding
