# Supplementary material for: The Promise and Limitations of Using Analogies to Improve Decision-Relevant Understanding of Climate Change
Source: PLoS One. 2017 Jan 30;12(1):e0171130. doi: 10.1371/journal.pone.0171130 (PMC5279784; doi:10.1371/journal.pone.0171130)
Supplement: S2 Table — (DOCX) [file pone.0171130.s002.docx]

**S2 Table. Analyses of variance (ANOVAs) testing main effects of condition on dependent measures (Study 1).**

|  | Control |  | MA |  | DA |  | TA |  |  |  |  |
| --- | --- | --- | --- | --- | --- | --- | --- | --- | --- | --- | --- |
| Dependent variable | Mean (SE) |  | Mean (SE) |  | Mean (SE) |  | Mean (SE) | *df* | *F* | *p* | *η^2^* |
| Helpfulness | 2.98 (0.12)^a^ |  | 3.75 (0.12)^b^ |  | 3.34 (0.12)^a,b^ |  | 3.41 (0.13)^a,b^ | 347 | 7.30 | < .001 | .06 |
| Literacy | 3.71 (0.10)^a^ |  | 4.18 (0.19)^a^ |  | 3.51 (0.20)^a^ |  | 3.80 (0.21)^a^ | 349 | 2.16 | .092 | .02 |
| Consensus | 75.13 (2.07)^a^ |  | 80.38 (2.02)^a^ |  | 77.84 (2.10)^a^ |  | 81.42 (2.27)^a^ | 349 | 1.75 | .156 | .01 |
| Element 1 | 4.09 (0.08)^a^ |  | 4.29 (0.08)^a^ |  | 4.11 (0.09)^a^ |  | 4.19 (0.09)^a^ | 349 | 1.21 | .306 | .01 |
| Element 2 | 4.14 (0.10)^a^ |  | 4.33 (0.10)^a^ |  | 4.07 (0.11)^a^ |  | 4.20 (0.11)^a^ | 346 | 1.19 | .313 | .01 |
| Element 3 | 3.99 (0.09)^a^ |  | 4.22 (0.09)^a^ |  | 4.04 (0.09)^a^ |  | 4.13 (0.10)^a^ | 349 | 1.27 | .283 | .01 |
| Element 4 | 3.99 (0.09)^a^ |  | 4.28 (0.09)^a^ |  | 4.08 (0.10)^a^ |  | 4.28 (0.10)^a^ | 349 | 2.33 | .074 | .01 |
| Element 5 | 3.95 (0.092)^a,b^ |  | 3.95 (0.09)^a,b^ |  | 3.99 (0.09)^a^ |  | 3.61 (0.10)^b^ | 348 | 3.12 | .026 | .03 |
| Element 6a | 3.76 (0.09)^a^ |  | 3.93 (0.08)^a^ |  | 3.98 (0.09)^a^ |  | 4.00 (0.09)^a^ | 349 | 1.53 | .206 | .01 |
| Element 6b | 3.01 (0.11)^a^ |  | 2.84 (0.11)^a^ |  | 3.00 (0.11)^a^ |  | 2.71 (0.12)^a^ | 348 | 1.52 | .210 | .01 |
| Element 7 | 3.61 (0.09)^a^ |  | 3.92 (0.09)^a,b^ |  | 3.92 (0.09)^a,b^ |  | 4.11 (0.10)^b^ | 349 | 4.69 | .003 | .04 |

*Note:* Means sharing the same superscript are not significantly different from each other (*p* < .05, pairwise comparisons using Sidak corrections)
