# Supplementary material for: The Promise and Limitations of Using Analogies to Improve Decision-Relevant Understanding of Climate Change
Source: PLoS One. 2017 Jan 30;12(1):e0171130. doi: 10.1371/journal.pone.0171130 (PMC5279784; doi:10.1371/journal.pone.0171130)
Supplement: S1 Appendix — (DOCX) [file pone.0171130.s003.docx]

**S1 Appendix**

**Experimental Conditions**

**Reader note*: Words in bold differed between conditions. Participants were not shown passage headings.

***Informational Control***

Large numbers of scientists and other people say that climate change is a global problem, one with serious consequences. But how should people think about this issue? It might help to think about it this way.

**The full consequences of climate change are not visible yet, but they can be expected to become very serious.** Climate experts say that they’ve run thousands of **scientific** tests over the past 50 years that show that the **planet is experiencing** **climate change.**

They know that as **climate change** progresses, we can expect to see increased numbers of severe **problems** (temperature and sea level rise, damaged ecosystems, etc.). They cannot be sure that each particular **problem** is due to climate change rather than some other cause, but they are very confident that **climate change** is present and getting worse. They are also confident that the main causes are human activities: fossil fuel use, the way people use land, and some other activities.

What will happen next? The scientists are certain that **climate change** is very hard to reverse. However, they aren’t certain about how fast it will progress or where and when to expect the most serious **problems.** There could be catastrophic results, especially in some places, but scientists aren’t yet confident about which very serious outcomes to expect, or how soon.

***Medical Analogy Condition***

Large numbers of scientists and other people say that climate change is a global problem, one with serious consequences. But how should people think about this issue? It might help to think about it this way.

**Imagine that Earth is a patient who has been diagnosed with a serious disease and that you are the patient’s (Earth’s) legal guardian**. Climate experts say that, **as with heart disease or cancer, the symptoms** of climate change are not **obvious in the early stages**, **but the disease is just as serious.** They say that they’ve run thousands of **diagnostic** tests over the past 50 years that show that the **patient has a disease: climate change.**

They know that as **the disease** progresses, we can expect to see increased numbers of severe **symptoms** (temperature and sea level rise, damaged ecosystems, etc.). They cannot be sure that each particular **symptom** is due to climate change rather than some other cause, but they are very confident that **the** **disease** is present and getting worse. They are also confident that the main causes are human activities: fossil fuel use, the way people use land, and some other activities.

What will happen next? The scientists are certain that **the disease** is very hard to reverse. However, they aren’t certain about how fast it will progress or where and when to expect the most serious **symptoms**. There could be catastrophic results, especially in some places, but scientists aren’t yet confident about which very serious outcomes to expect, or how soon.

What should be done? There are several **treatments** that get at the causes and can even reverse the **disease,** like reducing use of fossil fuels. Other **treatments** don’t treat causes, but they make it easier to live with **symptoms**, like building sea walls to reduce flood damage. But all the options have costs and risks. You want **treatments** that improve the **patient’s** chances at low cost and with limited side effects. You could take a wait-and-see approach and hope that nothing serious happens, but the longer **treatment** is postponed, the worse the **disease** will become.

***Disaster Preparedness Analogy Condition***

Large numbers of scientists and other people say that climate change is a global problem, one with serious consequences. But how should people think about this issue? It might help to think about it this way.

**Imagine that** **Earth is like a home that has to be protected against disasters like fire and flood.** Climate experts say that **you can’t be sure what the worst damage will be, but the consequences could be very serious.** They say that they’ve run thousands of **scientific** tests over the past 50 years that show that the **risks of climate change disasters are increasing**.

They know that as **climate change** progresses, we can expect to see increased numbers of severe **problems** (temperature and sea level rise, damaged ecosystems, etc.). **Disaster experts assess the risk to a house by looking at trends. Similarly, climate scientists** cannot be sure that each particular **disaster** is due to climate change rather than some other cause, but they are very confident that **the risk** is present and getting worse. They are also confident that the main causes are human activities: fossil fuel use, the way people use land, and some other activities.

What will happen next? The scientists are certain that **climate change** is very hard to reverse. However, they aren’t certain about how fast it will progress or where and when to expect the most serious **disasters.** There could be catastrophic results, especially in some places, but scientists aren’t yet confident about which very serious outcomes to expect, or how soon.

What should be done? There are several **responses** that get at the causes and can even reverse **the likelihood of disaster,** like reducing use of fossil fuels. **This would be similar to precautions like updating old wiring to prevent fires.** Other **actions** don’t treat causes, but they make it easier to live with **the effects**, like building sea walls to reduce flood damage. But all the options have costs and risks. You want **responses** that improve the **home’s** chances at low cost and with limited side effects. You could take a wait-and-see approach and hope that nothing serious happens, **but the longer you wait, the more likely it is that your home will suffer from a disaster.**

***Trial Analogy Condition***

Large numbers of scientists and other people say that climate change is a global problem, one with serious consequences. But how should people think about this issue? It might help to think about it this way.

**Imagine that you are a juror in a trial. You have to decide whether climate change is occurring and whether the defendant (human activity) is to blame.** Expert **witnesses** say that **even though the** **full consequences of climate change are not visible yet, they can be expected to become very serious.** They say **that climate experts have** run thousands of **scientific** tests over the past 50 years that show that the **planet is experiencing** **climate change.**

**The expert witnesses testify that** as **climate change** progresses, we can expect to see increased numbers of severe **problems** (temperature and sea level rise, damaged ecosystems, etc.).They **state** that they cannot be sure that each particular **problem** is due to climate change rather than some other cause, but they are very confident that **climate change** is present and getting worse. **They are** also confident that the main causes are human activities: fossil fuel use, the way people use land, and some other activities.

What will happen next? **The experts testify** that **climate change** is very hard to reverse. **They also say** there could be catastrophic results, especially in some places, but scientists aren’t yet confident about which very serious outcomes to expect, or how soon.

What should be done? **If you decide that human activity is causing climate change, you must consider what the remedy should be.** There are several **responses** that get at the causes and can even reverse **climate change**, like reducing use of fossil fuels. Other **responses** don’t treat causes, but they make it easier to live with **the predicted effects**, like building sea walls to reduce flood damage. But all the options have costs and risks. You want a **remedy that creates the best outcome for the community** at low cost and with limited side effects. You could also **decide that the defendant is not guilty and do nothing for now, but if you are wrong, the longer you wait, the more damage will be done.**
